# Spatial and Ecological Factors Modulate the Incidence of Anti-NMDAR Encephalitis—A Systematic Review

**DOI:** 10.3390/biomedicines11061525

**Published:** 2023-05-25

**Authors:** Agustí Alentorn, Giulia Berzero, Harry Alexopoulos, John Tzartos, Germán Reyes Botero, Andrea Morales Martínez, Sergio Muñiz-Castrillo, Alberto Vogrig, Bastien Joubert, Francisco A. García Jiménez, Dagoberto Cabrera, José Vladimir Tobon, Carolina Delgado, Patricio Sandoval, Mónica Troncoso, Lorna Galleguillos, Marine Giry, Marion Benazra, Isaias Hernández Verdin, Maëlle Dade, Géraldine Picard, Véronique Rogemond, Nicolas Weiss, Marinos C. Dalakas, Pierre-Yves Boëlle, Jean-Yves Delattre, Jérôme Honnorat, Dimitri Psimaras

**Affiliations:** 1 Department of Neurology 2 Mazarin, Hôpitaux Universitaires La Pitié Salpêtrière, Assistance Publique Hôpitaux de Paris, APHP, 75013 Paris, France; 2 Inserm U 1127, CNRS UMR 7225, Institut du Cerveau et de la Moelle épinière, ICM, Université Pierre-et-Marie-Curie, Sorbonnes Universités, 75005 Paris, France; 3 Neuroimmunology Unit, Department of Pathophysiology, Faculty of Medicine, National and Kapodistrian University of Athens, 11527 Athens, Greece; 4 1st Department of Neurology, Eginition Hospital, Medical School, National and Kapodistrian University of Athens, 72-74, Vas. Sofias Ave, 11528 Athens, Greece; 5 Department of Oncology, Neuro-Oncology Section, Hospital Pablo Tobón Uribe, Medellín 050010, Colombia; 6 Departments of Neurology and Neurosurgery, Hospital Clínico Universidad de Chile, Santiago 8380456, Chile; 7 French Reference Center on Paraneoplastic Neurological Syndromes, Hospices Civils de Lyon, Hôpital Neurologique, 69677 Bron, France; 8 Institut NeuroMyoGene INSERM U1217/CNRS UMR 5310, Université de Lyon, Université Claude Bernard Lyon 1, 69372 Lyon, France; 9 Department of Neurology, Faculty of Medicine, University of Antioquia, Carrera 51d N° 62-29, Medellín 050010, Colombia; 10 Department of Neurology, Hospital Universitario San Vicente Fundación, Calle 64N° 51d-154, Medellín 050010, Colombia; 11 Deparment of Neuropediatry, Hospital Universitario San Vicente Fundación, Calle 64N° 51d-154, Medellín 050010, Colombia; 12 Instituto Neurologico de Colombia, University of Antioquia, Medellin 050010, Colombia; 13 Department of Neurology, Faculty of Medicine, Pontificia Universidad Católica de Chile, Santiago 7820436, Chile; 14 Department of Pediatric Neurology, Hospital Clínico San Borja Arriarán, Facultad de Medicina, Campus Centro, Universidad de Chile, Santiago 7800003, Chile; 15 Department of Neuroimmunology, Clinica Davila, Santiago 8431657, Chile; 16 Department of Neurology, Neuro ICU, Hôpitaux Universitaires La Pitié Salpêtrière, Assistance Publique Hôpitaux de Paris, APHP, 75013 Paris, France; 17 INSERM, Sorbonne Université, Institut Pierre Louis d’Épidémiologie et de Santé Publique, 75012 Paris, France; 18 Centre de Compétence des Syndromes Neurologiques Paraneoplasiques et Encéphalites Autoimmunes, Groupe Hospitalier Pitié-Salpêtrière, 75013 Paris, France

**Keywords:** anti-NMDAR encephalitis, geoepidemiology, seasonality

## Abstract

Anti-NMDAR encephalitis has been associated with multiple antigenic triggers (i.e., ovarian teratomas, prodromal viral infections) but whether geographic, climatic, and environmental factors might influence disease risk has not been explored yet. We performed a systematic review and a meta-analysis of all published papers reporting the incidence of anti-NMDAR encephalitis in a definite country or region. We performed several multivariate spatial autocorrelation analyses to analyze the spatial variations in the incidence of anti-NMDA encephalitis depending on its geographical localization and temperature. Finally, we performed seasonal analyses in two original datasets from France and Greece and assessed the impact of temperature using an exposure-lag-response model in the French dataset. The reported incidence of anti-NMDAR encephalitis varied considerably among studies and countries, being higher in Oceania and South America (0.2 and 0.16 per 100,000 persons-year, respectively) compared to Europe and North America (0.06 per 100,000 persons-year) (*p* < 0.01). Different regression models confirmed a strong negative correlation with latitude (Pearson’s R = −0.88, *p* < 0.00001), with higher incidence in southern hemisphere countries far from the equator. Seasonal analyses showed a peak of cases during warm months. Exposure-lag-response models confirmed a positive correlation between extreme hot temperatures and the incidence of anti-NMDAR encephalitis in France (*p* = 0.03). Temperature analyses showed a significant association with higher mean temperatures and positive correlation with higher ultraviolet exposure worldwide. This study provides the first evidence that geographic and climatic factors including latitude, mean annual temperature, and ultraviolet exposure, might modify disease risk.

## 1. Introduction

The description of the *N*-Methyl-d-Aspartate receptor (NMDAR) encephalitis in 2007 [1] represented a paradigm shift in the field of neuroimmunology. Anti-NMDAR encephalitis is autoimmune encephalitis related to the presence of autoantibodies of the IgG1 subclass targeting the GluN1 subunit of the NMDAR, a glutamate receptor highly expressed on the surface of hippocampal neurons. Anti-NMDAR autoantibodies have demonstrated to be directly pathogenic, causing reversible synaptic dysfunction in neuronal cultures [2] and animal models [3]. 

Anti-NMDAR encephalitis primarily affects young females [4,5], one of the triggers being the presence of an underlying ovarian teratoma, which is detected in about half of the cases [4,5]. Preceding viral infections [5], not limited to herpes simplex [6] and Japanese encephalitis [7], represent additional triggers of the disease. Genetic factors such as HLA profile (HLA-I B*07:02 in European patients and HLA-II DRB1*16:02 in Chinese populations) have also been suggested to modulate disease risk [8,9], supporting the hypothesis that, similarly to several other autoimmune disorders, both genetic and environmental factors may concur to disease pathogenesis [10]. 

The possible impact of environmental factors such as latitude, sun exposure, and air pollution has not yet been evaluated in autoimmune encephalitis, although they have shown to heavily influence the risk and disease activity of multiple sclerosis [11,12], another immune-mediated disorder affecting the central nervous system. 

Herein, we performed a systematic review and a meta-analysis of the literature, including unpublished datasets from four additional countries, to assess the incidence of anti-NMDAR encephalitis in different countries, searching for elements suggesting an influence from geographic, climatic, and environmental factors. To strengthen our findings, we performed seasonal and climatic analyses on two original datasets from France and Greece.

## 2. Materials and Methods

The literature review was conducted and reported following PRISMA statements [13]. The PubMed (https://pubmed.ncbi.nlm.nih.gov/, accesed on 15 January 2020) and Google Scholar (https://scholar.google.com/, accessed on 15 January 2020) research was performed between 20 December 2019 and 15 January 2020 using the keywords ‘autoimmune encephalitis’ and ‘NMDA encephalitis’ in combination with each of the 177 country names included in the ISO list of world countries (Supplementary Table S1). No restriction was applied concerning language or year of publication. 

Two investigators (AA, GB) independently reviewed the articles retrieved from the research, extracting relevant information using a standardized data extraction sheet, as recommended by quality standards for reporting meta-analyses of observational studies in epidemiology [14]. The assessments performed separately by the two investigators were then cross-checked and, if any disagreement arose, a third reviewer (DP) was consulted to achieve a final decision.

To be included in the meta-analysis, studies needed to provide the number of incident cases or crude and/or age-specific incidence estimates for anti-NMDAR encephalitis, study period, and referring population. Studies reporting incidence estimates inferred from a subset of patients not representative of the whole anti-NMDAR encephalitis population (i.e., concerning intensive care unit or epileptic patients) were excluded. The list of the 68 studies [9,15,16,17,18,19,20,21,22,23,24,25,26,27,28,29,30,31,32,33,34,35,36,37,38,39,40,41,42,43,44,45,46,47,48,49,50,51,52,53,54,55,56,57,58,59,60,61,62,63,64,65,66,67,68,69,70,71,72,73,74,75,76,77,78,79,80,81,82] included in the meta-analysis is given in the Supplementary Data.

Literature data were complemented by unpublished data on the incidence of NMDAR encephalitis in Colombia, Chile, France, and Greece, collected by coauthors actively working in these countries. This allowed having information on the incidence of anti-NMDAR encephalitis in three countries, with no literature data available (Colombia, Chile, and Greece). This study was approved by the local ethics committee of the Pitié Salpêtrière Hospital and informed consent was waived (reference CPP SUD-EST II).

Two unpublished datasets of patients diagnosed with “definite” anti-NMDAR encephalitis according to the 2016 criteria [83], one from Greece and one from France, were used as exploratory datasets for additional analyses on seasonal and climatic trends. Individual data on Greek patients’ dataset were collected retrospectively during the period 2010–2019 from two diagnostic neuroimmune laboratories considered as nation-wide referral centers. Individual data on French patients were drawn from the database of the National Reference Centre for Paraneoplastic Neurological Syndromes and refer to the period 2008–2018. 

Incidence rates were calculated using the number of incident cases per year over the referring population, assuming that reference populations were stable throughout the study period. We calculated an age and sex standardized incidence as directly standardized rates (DSR) using a gamma distribution [84] with the World Health Organization standard population with five-year intervals. We also obtained the female/male ratio with the 95% confidence interval (CI) using the Wald normal approximation and considering counts and person-year [85].

Overall incidence estimates were calculated using both fixed and random-effects models, weighted for inverse variance following DerSimonian’s method [86]. Heterogeneity between studies was assessed using a chi-square test (Cochran’s Q statistic) and quantified using the I² statistic [87].

Publication bias was evaluated with the aid of a funnel plot, the asymmetry of which was assessed with the Egger’s test [88]. The differences between different subgroups within the meta-analysis were assessed using different meta-regression models (Supplementary Methods).

Spatial autocorrelation refers to the correlation of a variable with itself in space. In our case, the variable was the incidence of anti-NMDAR encephalitis: a positive spatial autocorrelation existed if high incidence was associated with high incidence in neighboring countries, while a negative spatial autocorrelation existed if low incidence was associated with high incidence in neighboring countries. Global spatial autocorrelation was assessed using the Moran’s I and the Geary’s C indexes. 

We assessed different multivariate spatial regression models (i.e., geographically weighted regression, ordinary least square regression, generalized additional model, and conditional and simultaneously autoregressive models) adjusting with the mean temperature of each country to further characterize in a multivariate model the spatial correlation of anti-NMDAR encephalitis with its spatial distribution. We selected the model with the best performance according to the minimum Akaike Information Criteria (AIC). From these models, we obtained the local R^2^ that were mapped. In addition, these models provided a prediction of anti-NMDAR encephalitis at a worldwide level.

### 2.1. Correlating the Incidence of Anti-NMDAR Encephalitis with Different Climatic, Environmental, and Demographic Factors

We performed several linear regressions to correlate the incidence of anti-NMDAR encephalitis with different environmental, climatologic, or demographic features. The degree of correlation was assessed using the coefficient R of Pearson’s correlation. Demographic features included the urban population percentage or the socio-demographic index (SDI) [89]. Climatic and environmental variables included mean annual temperature, particulate matter air pollution (PM_2.5_) exposure, the median CO_2_ emissions per country, and the ultraviolet exposure in each included country (Supplementary Methods).

We performed an exposure-lag-response regression between the number of anti-NMDAR encephalitis cases and the temperature in France and in Ile-de-France, as previously described [90] (Supplementary Methods).

### 2.2. Seasonal and Monthly Trends

After comparing the accuracy of different seasonal and non-seasonal models, we used the X13-Seasonal Extraction in Autoregressive Integrated Moving Average (ARIMA) Time-Series (SEATS)-ARIMA algorithm [91] in the French dataset, and the Seasonal and Trend decomposition using locally weighted running line smoother (LOESS), STL [92] in the Greek dataset, to assess temporal trends (Supplementary Methods). 

We compared average monthly counts of anti-NMDAR encephalitis, using a Quasi-Poisson regression, with the number of cases as the outcome, month as the sole predictor (with February as baseline), and the log of the population as an offset (Supplementary Methods).

All statistical analyses were performed using the software “R” (version 4.0.1). The threshold for statistical significance was *p* < 0.05, all tests were bilateral. Details from the different version of R packages as well as the R scripts, datasets, and the methodological details used to reproduce the vast majority of the results are provided in the Supplementary Methods and can be found at https://osf.io/u5hjf/?view_only=bb4ed5d417b6410c8c3a9ebad81bee09, accessed on 15 October 2022.

## 3. Results

### 3.1. Literature Meta-Analysis

**Figure 1:**
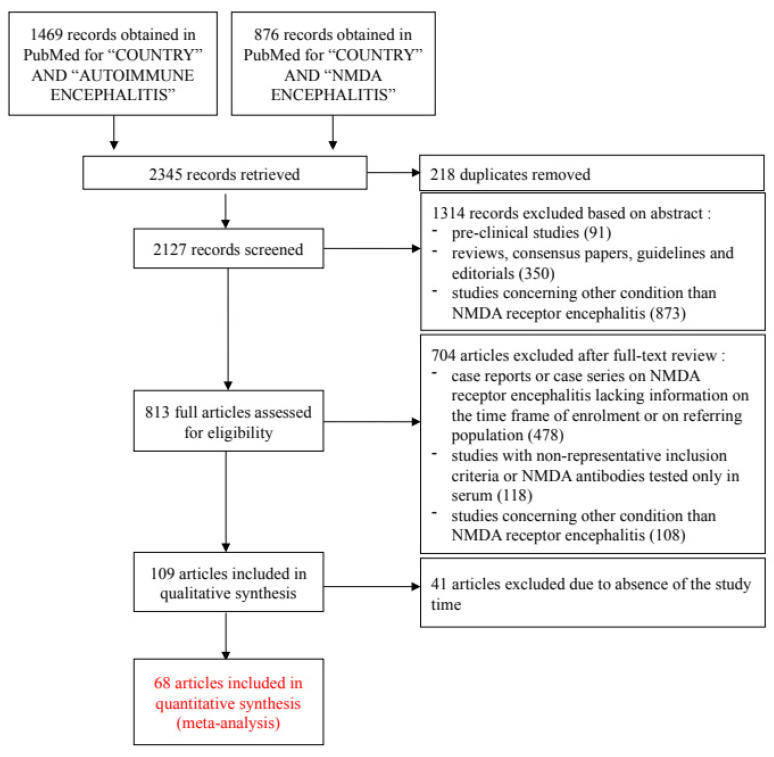
Flow chart for study selection according to the PRISMA guidelines.

Our research strategy yielded 2127 unique records in PubMed. After a systematic process of exclusion (Figure 1), we were left with 68 articles that, with the addition of four unpublished studies, provided information on the incidence of anti-NMDAR encephalitis in 30 different countries, Supplementary Table S2.

**Figure 2:**
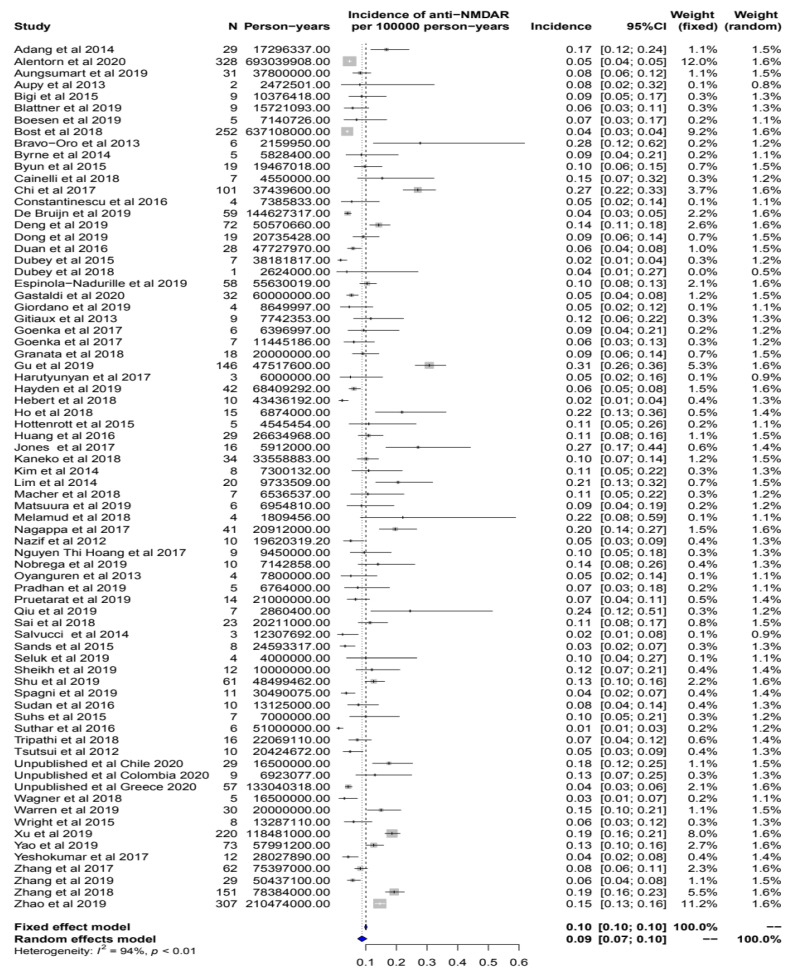
Forest plot summarizing the estimates for the population-based incidence of anti-NMDAR encephalitis within the different studies included in the meta-analysis. Summary is expressed as the number of cases of anti-NMDAR encephalitis per 100,000 inhabitants-year. An I² value (statistical heterogeneity) of 94% indicates high variability.

The crude population-based incidences of anti-NMDAR encephalitis within the different studies included in the meta-analysis are summarized in the Forest plot in Figure 2. Pooling together the data from all the studies, the crude overall incidence estimate for anti-NMDAR encephalitis, calculated using a random effect model, was 0.09 per 100,000 inhabitants-year (95% CI: 0.07–0.10). The value of statistical heterogeneity for this analysis was high (I² = 94%), reflecting how the crude incidence of anti-NMDAR encephalitis varied across studies (from 0.01 to 0.31 cases per 100,000 inhabitants-year). The Funnel plot (Supplementary Figure S2) revealed a relatively symmetrical distribution of the studies, with Egger’s bias test *p* = 0.3, suggesting a non-significant asymmetry of studies.

### 3.2. Unpublished Data

Literature data were complemented by unpublished data on the incidence of NMDAR encephalitis in Colombia, Chile, France, and Greece, collected by coauthors actively working in these countries. This allowed having information on the incidence of anti-NMDAR encephalitis in three countries, with no literature data available (Colombia, Chile, and Greece). 

### 3.3. The Incidence of Anti-NMDAR Encephalitis Differs between Continents

**Figure 3:**
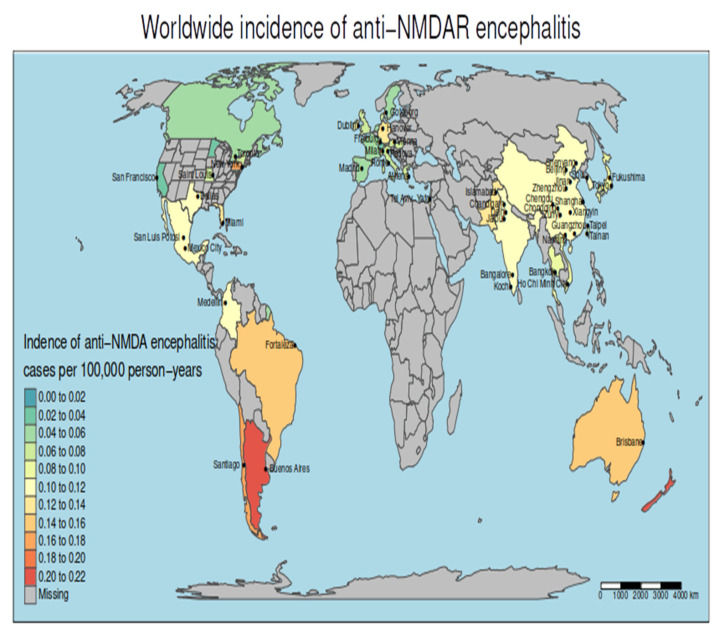
Worldwide distribution of anti-NMDAR encephalitis using the data from the systematic review and meta-analysis. A city name appears when the study considered for that country used data from that city and its referral area while, when no city is indicated, studies were performed at a country-wide level.

We graphically represented crude incidence rates in the countries included in our meta-analysis in a world map (Figure 3). The crude incidence of anti-NMDAR encephalitis differed based on the continent, varying from 0.06 per 100,000 inhabitants-year (95% CI 0.05–0.07) in Europe (Supplementary Figure S2) and 0.06 (0.04–0.09) in North America (Supplementary Figure S3) to 0.11 per 100,000 inhabitants-year (0.09–0.13) in Asia (Supplementary Figure S4), 0.16 (0.12–0.21) in South America (Supplementary Figure S5), and 0.2 (0.11–0.35) in Oceania (including Australia and New Zealand) (Supplementary Figure S6). According to different meta-regressions, the incidence of anti-NMDAR encephalitis was significantly lower in Europe than in South America, Oceania and Asia (*p* < 0.0001, *p* < 0.0001 and *p* = 0.001, respectively), while it was very similar to North America (*p* = 0.9). Similarly, South America, Asia, and Oceania countries, had a higher incidence than North America countries, *p* = 0.01, *p* < 0.001, and *p* = 0.02, respectively. Finally, Asia showed an intermediate incidence of 0.11 per 100 000 inhabitants-year (0.09–0.13), with no significant differences compared to South America (*p* = 0.2) or Oceania (*p* = 0.5). 

### 3.4. Geographical Clusters of Higher and Lower Incidence

Global spatial autocorrelation analyses showed a moderate but significant correlation between the incidence of anti-NMDAR encephalitis and geography (Moran’s I = 0.23, *p* < 0.00001; Geary’s C = 0.39, *p* < 0.00001). 

### 3.5. Multivariate Spatial Analyses: The Impact of Temperature and Latitude

We compared several multivariate spatial regression models (Supplementary Methods) and we selected the GWR model because it had the highest adjusted R² (0.89) and the lowest AIC (−841). The GWR was chosen to assess spatial autocorrelation (Supplementary Figure S7A) in a multivariate model, adjusting by the mean annual temperature of each country (Supplementary Figure S7B). We used the GWR to calculate local R² for each country and obtained high local R^2^ values (0.75–1), reflecting high goodness of fit of a model, in North America, in Oceania, and most European countries (Supplementary Figure S7C). The GWR was then used to produce a map of the predicted probability of anti-NMDAR encephalitis, which showed a higher risk for southern hemisphere countries (Supplementary Figure S7D). 

**Figure 4:**
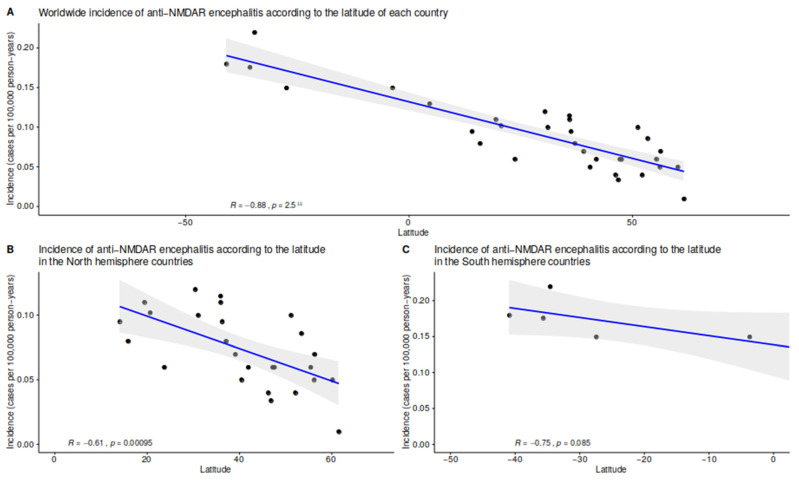
Linear regressions assessing the association between the latitude of the country and the incidence of anti-NMDAR encephalitis from the meta-analysis, worldwide (**A**–**C**). The grey band around the regression line represents the 95% CI. The R is estimated using the Pearson correlation.

In addition to GWR, multiple linear regression models were used to explore the correlation between the incidence of anti-NMDAR encephalitis and geographic, climatic, environmental, and demographic factors. Interestingly, we observed a strong negative correlation between latitude and the incidence of anti-NMDAR encephalitis (R = −0.88, *p* < 0.00001) (Figure 4), the incidence of anti-NMDAR encephalitis increasing progressively from the North of Europe to Argentina. The incidence of anti-NMDAR encephalitis showed a positive correlation with mean annual temperature (R = 0.45, *p* = 0.01) (Supplementary Figure S8) and ultraviolet exposure (R = 0.46, *p* = 0.02) (Supplementary Figure S9), increasing with higher mean annual temperatures and higher ultraviolet exposure. In northern hemisphere countries, the incidence of anti-NMDAR encephalitis showed an inverse correlation with CO_2_ emissions (R = −0.5, *p* = 0.008, Supplementary Figure S10), particulate matter air pollution PM2.5 (R = 0.55, *p* = 0.005) (Supplementary Figure S11), urban population percentage (R = −0.4, *p* = 0.03) (Supplementary Figure S12), and SDI (R = −0.4, *p* = 0.02) (Supplementary Figure S13). These observations did not apply to southern hemisphere countries, where most *p*-values did not reach statistical significance. 

### 3.6. Spatial and Temporal Analyses on the French and Greek Dataset

To better assess the impact of some climatic variables on the incidence of anti-NMDAR encephalitis, we analyzed two unpublished cohorts, one from France (*n* = 329, 328 with age available) and one from Greece (*n* = 57). The crude and standardized incidences of anti-NMDAR encephalitis in the French and Greek datasets are provided in Supplementary Tables S3 and S4. We represented the female/male ratio according to a five-year interval in two datasets with a higher proportion of females. Female predominance was stronger in younger patients (Supplementary Figure S14). The frequency of the different tumors associated with anti-NMDAR encephalitis is described in Supplementary Figure S15.

**Figure 5:**
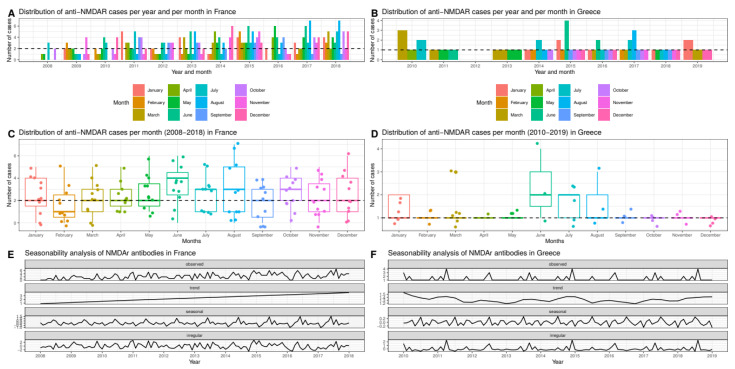
Temporal distribution of anti-NMDAR encephalitis cases in France (2008–2018) (panel **A**,**C**,**E**) and Greece (2010–2019) (panel **B**,**D**,**F**). (Panel **A**,**B**) show the aggregated number of cases per year and month; (panel **C**,**D**) show the number of cases per month (dotted lines in both panels indicate median values); (panel **E**,**F**) show long-term trends and seasonality.

These two datasets were used for temporal distribution analyses. In the French dataset, the median number of cases was 2 per month, with a recurrent lower number of cases in February and a recurrent higher number of cases in June and August (Figure 5, panel C). In the Greek dataset, where the median number of cases was 1 per month, this trend was far less evident (Figure 5, panel D). To better circumstantiate monthly variations, we performed a Quasi-Poisson regression using February as the month of reference. The Quasi-Poisson regression disclosed a significant higher number of cases in June in both countries, with a relative risk (RR) of 2.1 (95% CI 1.2–4) and *p* = 0.02 in France and a RR of 2.3 (1.2–4.7) and *p* = 0.02 in the Greek dataset. 

These findings prompted us to perform more in-depth seasonal analyses using the ARIMA model (French dataset) and the STL model (Greek dataset), details are provided in the Supplementary Methods. These models confirmed the existence of a seasonal trend in both countries, with a recurrent peak of cases during summer (Figure 5, panel E and F). The same models disclosed a progressive increase in the number of cases of anti-NMDAR encephalitis diagnosed in France over the study interval (2008–2018) (Figure 5, panel E) that was not observed in Greece, where the yearly number of cases remained relatively constant from 2010 to 2019 (Figure 5, panel F). 

**Figure 6:**
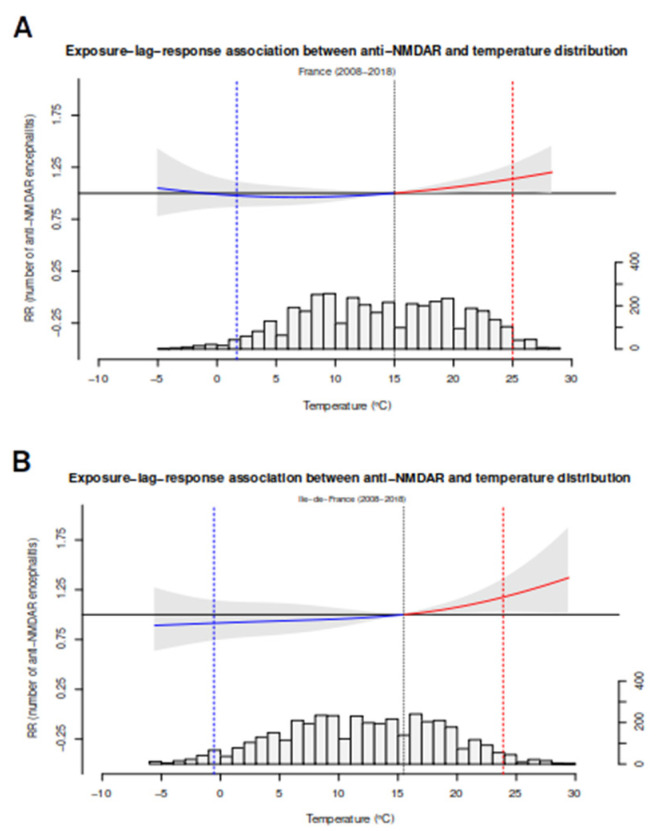
Exposure-lag-association model between anti-NMDAR encephalitis and the temperature in the Ile-de-France region (panel **A**) and all France (panel **B**). Exposure–response associations as best linear unbiased prediction (with 95% CI, shaded grey), with related temperature distributions. Solid grey lines are minimum incidence temperatures and dotted lines are the 2·5th and 97·5th percentiles. Note that in both models, the grey area is slightly above the horizontal line (RR = 1), showing a significant association. RR = relative risk.

Based on the observation that the incidence of anti-NMDAR encephalitis seemed to increase during warm months, we used the French dataset to perform temperature analyses. Tlag-he impact of temperature on the incidence of anti-NMDAR encephalitis was assessed using a lag-response association model. Both in the overall dataset (*n* = 328) and the subset of patients from the Île-de-France region (*n* = 115), which was the region with more cases, we observed a significant association between hot temperatures and the crude incidence of anti-NMDAR encephalitis in the overall dataset, RR 1.2 [1.02–1.4, *p* = 0.03, and also in Île-de-France, RR 1.15 [1.02–1.55] *p* = 0.04 (Figure 6, panel A and B), providing additional evidence in support of this association. This model was used to estimate the quantitative impact of temperature on the incidence of anti-NMDAR encephalitis. We found that high temperatures accounted for approximately 15% (CI 0.7–27%) of cases of anti-NMDAR encephalitis. The Greek dataset was not analyzed using this approach due to the limited number of patients. 

## 4. Discussion

In this systematic review and meta-analysis, we explored the association between the incidence of anti-NMDAR encephalitis and several geographic, climatic, and environmental factors. We found that the incidence of anti-NMDAR encephalitis strongly correlated with latitude, mean annual temperature, and ultraviolet exposure. We identified a seasonal distribution, with a peak of cases during warm months and a correlation with extreme hot temperatures. The results were not unanticipated, as several autoimmune diseases show a strong geographical distribution [93,94]. The best paradigm in neurology is multiple sclerosis, which has a higher incidence in countries far from the equator and lower incidence in countries near the equator [95,96]. This phenomenon has been attributed to mean vitamin D levels, which are dependent on sun exposure, and decrease as the distance from the equator increases, exposing to a higher disease risk [93]. Conversely, our meta-analysis suggests that ultraviolet exposure might influence the risk of anti-NMDAR encephalitis, the geographical distribution observed for anti-NMDAR encephalitis differs from the one depicted for multiple sclerosis, suggesting that additional climatic and environmental factors might be implicated. 

Similarly to sun exposure, mean annual temperature has shown to modulate the incidence of multiple sclerosis [97] and other autoimmune disorders [98] and might represent one of the factors responsible for the geographical gradient observed in our meta-analysis. Intriguingly, the exposure-lag-associated study pinpointed a non-linear association between hot temperatures and the incidence of anti-NMDAR encephalitis (Figure 6). Interestingly, this type of non-linear association with temperature and other health conditions has been previously described [90]. 

Consistently with temperature analysis, we observed that anti-NMDAR encephalitis displayed a seasonal pattern, with a higher number of cases during warm months. It should be noted that in the Greek dataset, with a limited number of cases, there was a great number of cases during June, July and August (Figure 5D). A similar observation was previously reported in a small study conducted in the United States on pediatric patients [15]; however, multivariate analyses or seasonal modelling were not included. Although, a higher peak of cases during summer could simply reflect higher seasonal temperatures, other factors that have not been taken into account in the present study, such as recurrent viral epidemics, might also be implicated. Some studies on infectious encephalitis, including herpes simplex virus encephalitis, conducted in western countries have pinpointed a higher incidence of hospital admissions during summer [99,100], while others have failed to disclose any significant seasonal pattern [101]. Besides virus commonly responsible for encephalitis [6,7], other non-neurotropic viruses display a seasonal pattern and might be responsible for the higher number of cases of anti-NMDAR encephalitis.

This study has several limitations related to the limited data available in the literature on this rare disorder. Most of the studies included in the meta-analysis were retrospective and, as such, potentially affected by referral, selection, and misclassification biases. In addition, we did not have patient individual data for most of the studies limiting some of analysis, for example, the possibility to analyze the seasonality at worldwide level or the potential impact of UV on the incidence of anti-NMDAR encephalitis and we could not include age and gender adjustment, due to this limitation. The differences in age composition rendered crude incidence estimates not directly comparable between populations. Data on the incidence of anti-NMDAR encephalitis were unavailable for many world countries, mainly in the southern hemisphere, and completely missing for Africa. Therefore, our results should be interpreted with caution when considering the data of southern hemisphere, including the association between NMDAR encephalitis incidence and ultraviolet radiation exposure and the mean annual temperature. 

The impact of factors such as nutrition and lifestyle could not be assessed in our meta-analysis, although these likely represent important elements, as demonstrated for other autoimmune disorders [102]. However, we used the Socio-Demographic Index (SDI) similar factors. This is a summary measure of a geography’s socio-demographic development. It is based on average income per person, educational attainment, and total fertility rate (TFR) (Supplementary Methods and Supplementary Figure S13). Studies assessing the changes in disease risk as a consequence of changes in the place of living, as performed in patients with multiple sclerosis [103], might help to assess the weight of environmental factors and living habits on disease risk.

Another factor that could not be assessed in our meta-analysis is the genetic risk, which could indeed contribute to the variations in the incidence of anti-NMDAR encephalitis among countries and populations. Studies on genetic risk factors are to date limited to European and Chinese patients [8,9], and only further studies in low- and high-risk populations will elucidate the relative weight of genetics. 

To sum up, this study provides the first evidence that geographic and climatic factors might modulate the risk of anti-NMDAR encephalitis, paving the way to a broader range of explorations on the impact of environmental factors in the pathogenesis of this rare disease. The approach described in this study could be applied to other types of autoimmune encephalitis, clarifying if other entities within this spectrum share similar risk factors. 

## Data Availability

The data and R scripts as well as the methodological details used to reproduce the vast majority of the results are provided in the Supplementary Methods.

## Supplementary Materials

The following are available online at https://www.mdpi.com/article/10.3390/biomedicines11061525/s1.
