# Supplementary material for: Spatial and Ecological Factors Modulate the Incidence of Anti-NMDAR Encephalitis—A Systematic Review
Source: Biomedicines. 2023 May 25;11(6):1525. doi: 10.3390/biomedicines11061525 (PMC10295747; doi:10.3390/biomedicines11061525)
Supplement: Supplementary file 1 [file biomedicines-11-01525-s001.zip › biomedicines-1899343-supplementary.pdf]

Table S1. The list of countries used in the meta-analysis research International Organization for Standardization (ISO).

ISO	Country
AFG	Afghanistan
AGO	Angola
ALB	Albania
ARE	United Arab Emirates
ARG	Argentina
ARM	Armenia
ATA	Antarctica
ATF	Fr. S. Antarctic Lands
AUS	Australia
AUT	Austria
AZE	Azerbaijan
BDI	Burundi
BEL	Belgium
BEN	Benin
BFA	Burkina Faso
BGD	Bangladesh
BGR	Bulgaria
BHS	Bahamas
BIH	Bosnia and Herz.
BLR	Belarus
BLZ	Belize
BOL	Bolivia
BRA	Brazil
BRN	Brunei
BTN	Bhutan
BWA	Botswana
CAF	Central African Rep.
CAN	Canada
CHE	Switzerland
CHL	Chile
CHN	China
CIV	Cote d'Ivoire
CMR	Cameroon
COD	Dem. Rep. Congo
COG	Congo
COL	Colombia
CRI	Costa Rica
CUB	Cuba
XTX	N. Cyprus
CYP	Cyprus
CZE	Czech Rep.
DEU	Germany
DJI	Djibouti
DNK	Denmark
DOM	Dominican Rep.
DZA	Algeria
ECU	Ecuador

EGY	Egypt
ERI	Eritrea
ESP	Spain
EST	Estonia
ETH	Ethiopia
FIN	Finland
FJI	Fiji
FLK	Falkland Is.
FRA	France
GAB	Gabon
GBR	United Kingdom
GEO	Georgia
GHA	Ghana
GIN	Guinea
GMB	Gambia
GNB	Guinea-Bissau
GNQ	Eq. Guinea
GRC	Greece
GRL	Greenland
GTM	Guatemala
GUY	Guyana
HND	Honduras
HRV	Croatia
HTI	Haiti
HUN	Hungary
IDN	Indonesia
IND	India
IRL	Ireland
IRN	Iran
IRQ	Iraq
ISL	Iceland
ISR	Israel
ITA	Italy
JAM	Jamaica
JOR	Jordan
JPN	Japan
KAZ	Kazakhstan
KEN	Kenya
KGZ	Kyrgyzstan
KHM	Cambodia
KOR	Korea
UNK	Kosovo
KWT	Kuwait
LAO	Lao PDR
LBN	Lebanon
LBR	Liberia
LBY	Libya
LKA	Sri Lanka
LSO	Lesotho
LTU	Lithuania
LUX	Luxembourg
LVA	Latvia

MAR	Morocco
MDA	Moldova
MDG	Madagascar
MEX	Mexico
MKD	Macedonia
MLI	Mali
MMR	Myanmar
MNE	Montenegro
MNG	Mongolia
MOZ	Mozambique
MRT	Mauritania
MWI	Malawi
MYS	Malaysia
NAM	Namibia
NCL	New Caledonia
NER	Niger
NGA	Nigeria
NIC	Nicaragua
NLD	Netherlands
NOR	Norway
NPL	Nepal
NZL	New Zealand
OMN	Oman
PAK	Pakistan
PAN	Panama
PER	Peru
PHL	Philippines
PNG	Papua New Guinea
POL	Poland
PRI	Puerto Rico
PRK	Dem. Rep. Korea
PRT	Portugal
PRY	Paraguay
PSE	Palestine
QAT	Qatar
ROU	Romania
RUS	Russia
RWA	Rwanda
ESH	W. Sahara
SAU	Saudi Arabia
SDN	Sudan
SSD	S. Sudan
SEN	Senegal
SLB	Solomon Is.
SLE	Sierra Leone
SLV	El Salvador
SOL	Somaliland
SOM	Somalia
SRB	Serbia
SUR	Suriname
SVK	Slovakia
SVN	Slovenia

SWE	Sweden
SWZ	Swaziland
SYR	Syria
TCD	Chad
TGO	Togo
THA	Thailand
TJK	Tajikistan
TKM	Turkmenistan
TLS	Timor-Leste
TTO	Trinidad and Tobago
TUN	Tunisia
TUR	Turkey
TWN	Taiwan
TZA	Tanzania
UGA	Uganda
UKR	Ukraine
URY	Uruguay
USA	United States
UZB	Uzbekistan
VEN	Venezuela
VNM	Vietnam
VUT	Vanuatu
YEM	Yemen
ZAF	South Africa
ZMB	Zambia
ZWE	Zimbabwe

Table S2. Details about the studies included in the meta-analysis.

Study	number_NMDA	Population_years	Country
Adang et al 2014	29	17296337	USA
Alentorn et al 2020	328	693039908	France
Aungsumart et al 2019	31	37800000	Thailand
Aupy et al 2013	2	2472501	France
Bigi et al 2015	9	10376418	Canada
Blattner et al 2019	9	15721093	USA
Boesen et al 2019	5	7140726	Denmark
Bost et al 2018	252	637108000	France
Bravo-Oro et al 2013	6	2159950	Mexico
Byrne et al 2014	5	5828400	Ireland
Byun et al 2015	19	19467018	Korea
Cainelli et al 2018	7	4550000	Italy
Chi et al 2017	101	37439600	China
Constantinescu et al 2016	4	7385833	Sweden
De Bruijn et al 2019	59	144627317	Netherlands
Deng et al 2019	72	50570660	China
Dong et al 2019	19	20735428	China
Duan et al 2016	28	47727970	Taiwan
Dubey et al 2015	7	38181817	USA
Dubey et al 2018	1	2624000	USA
Espinola-Nadurille et al 2019	58	55630019	Mexico
Gastaldi et al 2020	32	60000000	Italy
Giordano et al 2019	4	8649997	Italy

Gitiaux et al 2013	9	7742353	France
Goenka et al 2017	6	6396997	India
Goenka et al 2017	7	11445186	USA
Granata et al 2018	18	20000000	Italy
Gu et al 2019	146	47517600	China
Harutyunyan et al 2017	3	6000000	Austria
Hayden et al 2019	42	68409292	Hungary
Hebert et al 2018	10	43436192	Canada
Ho et al 2018	15	6874000	China
Hottenrott et al 2015	5	4545454	Germany
Huang et al 2016	29	26634968	China
Jones et al 2017	16	5912000	New Zealand
Kaneko et al 2018	34	33558883	Japan
Kim et al 2014	8	7300132	Korea
Lim et al 2014	20	9733509	Korea
Macher et al 2018	7	6536537	Austria
Matsuura et al 2019	6	6954810	Japan
Melamud et al 2018	4	1809456	Argentina
Nagappa et al 2017	41	20912000	India
Nazif et al 2012	10	19620319.2	USA
Nguyen Thi Hoang et al 2017	9	9450000	Vietnam
Nobrega et al 2019	10	7142858	Brazil
Oyanguren et al 2013	4	7800000	Spain
Pradhan et al 2019	5	6764000	India
Pruetarat et al 2019	14	21000000	Thailand
Qiu et al 2019	7	2860400	China
Sai et al 2018	23	20211000	China
Salvucci et al 2014	3	12307692	USA
Sands et al 2015	8	24593317	USA
Seluk et al 2019	4	4000000	Israel
Sheikh et al 2019	12	10000000	Pakistan
Shu et al 2019	61	48499462	China
Spagni et al 2019	11	30490075	Italy
Sudan et al 2016	10	13125000	India
Suhs et al 2015	7	7000000	Germany
Suthar et al 2016	6	51000000	India
Tripathi et al 2018	16	22069110	India
Tsutsui et al 2012	10	20424672	Japan
Unpublished et al Chile 2020	29	16500000	Chile
Unpublished et al Colombia 2020	9	6923077	Colombia
Unpublished et al Greece 2020	57	133040318	Greece
Wagner et al 2018	5	16500000	Austria
Warren et al 2019	30	20000000	Australia
Wright et al 2015	8	13287110	UK
Xu et al 2019	220	118481000	China
Yao et al 2019	73	57991200	China
Yeshokumar et al 2017	12	28027890	USA
Zhang et al 2017	62	75397000	China
Zhang et al 2019	29	50437100	China
Zhang et al 2018	151	78384000	China
Zhao et al 2019	307	210474000	China

Table S3. Crude and standardized incidence according to 5-year age groups in France (2008–2018). Std: standardized; CI: confidence interval

Age groups	Num NMDA encephalitis	Person-years	Crude Incidence (per 100,000)	95% CI (crude)	Std Incidence (per 100,000)	95% CI (Std)
0-4	27	41864867	0.0645	0.04-0.09	0.05	0.02-0.09
5- 9	30	45749319	0.0656	0.04-0.09	0.06	0.03-0.1
10-14	26	45833865	0.0567	0.04-0.08	0.03	0.01-0.07
15-19	55	45421849	0.1211	0.09-0.16	0.03	0.01-0.06
20-24	65	40862888	0.1591	0.12-0.2	0.05	0.02-0.09
25-29	48	42535284	0.1128	0.08-0.15	0.03	0.008-0.06
30-34	21	44491128	0.0472	0.03-0.07	0.01	0.003-0.04
35-39	12	46520144	0.0258	0.01-0.04	0.01	0.001-0.03
40-44	11	46268926	0.0238	0.01-0.04	0.01	0.003-0.04
45-49	10	49941232	0.02	0.01-0.04	0.01	0.002-0.03
50-54	6	49387041	0.0121	0.004-0.03	0.004	0.0001-0.02
55-59	2	47152688	0.0042	0.0005-0.01	0.004	0.0001-0.02
60-64	6	44689337	0.0134	0.005-0.03	0.009	0.001-0.03
65-69	3	43406154	0.0069	0.001-0.02	0.005	0.0001-0.03
70-74	3	33406329	0.009	0.002-0.03	0.006	0.0002-0.04
75-79	2	13184974	0.0152	0.002-0.06	0.01	0.002-0.06
80-84	1	12323883	0.0081	0.0002-0.04	0.008	0.0002-0.04
Overall	328	693039908	Overall	0.03-0.07	0.04	0.03-0.07

Table S4. Crude and standardized incidence according to 5-year age groups in Greece (2010–2019). Std: standardized; CI: confidence interval,

Age groups	Num NMDA encephalitis	Person-years	Crude Incidence (per 100,000)	95% CI (Crude)	Std Incidence (per 100,000)	95% CI (Std)
0-4	8	10816286	0.074	0.032-0.14	0.0356	0.004-0.13
5 – 9	8	10167308.84	0.0787	0.034-0.155	0.0755	0.02-0.19
10-14	5	4975491.56	0.1005	0.033-0.23	0.1005	0.03-0.23

15-19	8	11032611.72	0.0725	0.031-0.14	0.034	0.004-0.12
20-24	5	12546891.76	0.0399	0.01-0.09	0.0303	0.004-0.11
25-29	6	14493823.24	0.0414	0.01-0.09	0.0132	0.003-0.07
30-34	5	16440754.72	0.0304	0.01-0.07	0.0234	0.003-0.08
35-39	3	7895888.78	0.038	0.008-0.11	0.038	0.008-0.11
40-44	2	8220377.36	0.0243	0.003-0.09	0.0243	0.003-0.09
45-49	1	7463237.34	0.0134	0.0003-0.07	0.0134	0.0003-0.07
55-59	2	6597934.46	0.0303	0.004-0.11	0.0303	0.004-0.11
60-64	2	12546891.76	0.0159	0.002-0.06	0.0162	0.0004-0.09
70-74	1	5840794.44	0.0171	0.0004-0.09	0.0171	0.0004-0.09
80-84	1	4002025.82	0.025	0.0006-0.14	0.025	0.0006-0.14
Overall	57	133040317.8	0.04	0.01-0.11	0.03	0.006-0.12

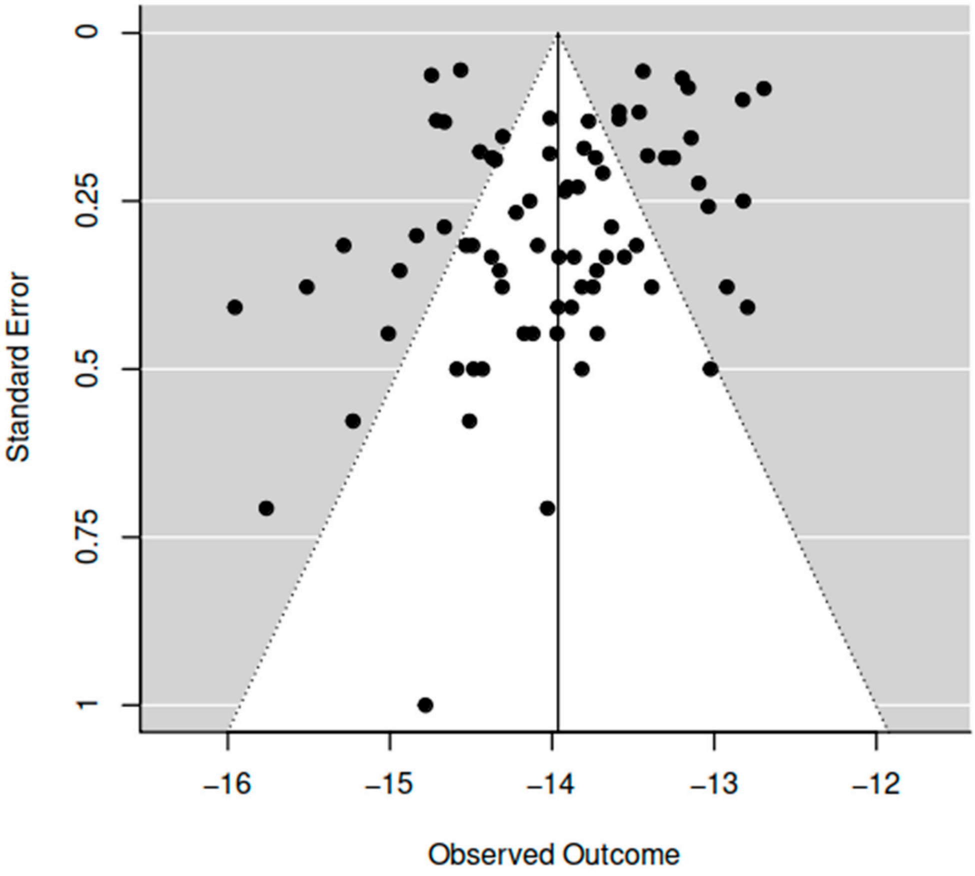

Figure S1.Funnel plot, showing a relative symmetrical distribution of the studies but lower representation of small sized studies (bottom part of the plot)

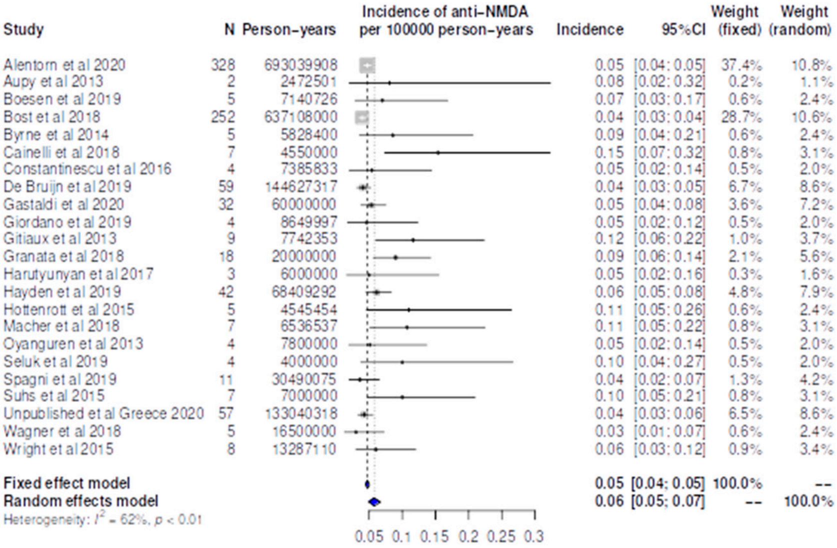

Figure S2. Forest plot summarizing the estimates for the population-based incidence of anti-NMDAR encephalitis in European countries included in the meta-analysis

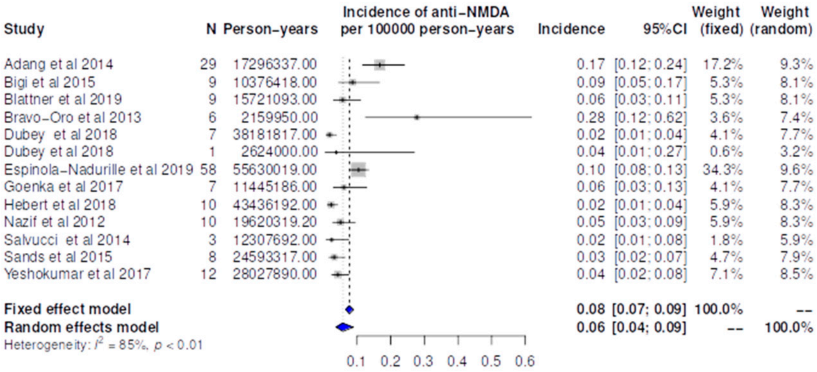

Figure S3. Forest plot summarizing the estimates for the population-based incidence of anti-NMDAR encephalitis in North American countries included in the meta-analysis

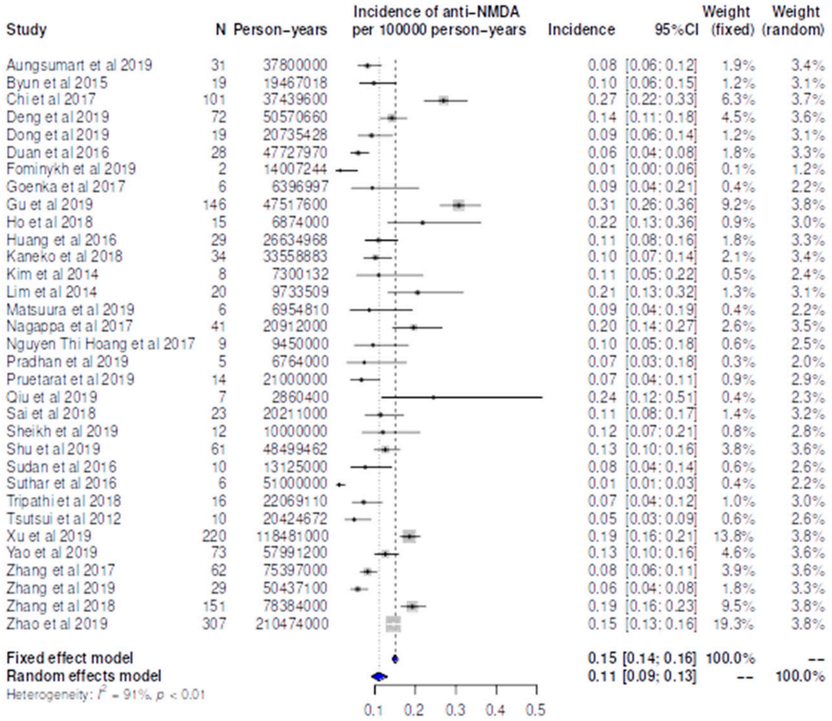

Figure S4. Forest plot summarizing the estimates for the population-based incidence of anti-NMDAR encephalitis in Asian countries included in the meta-analysis

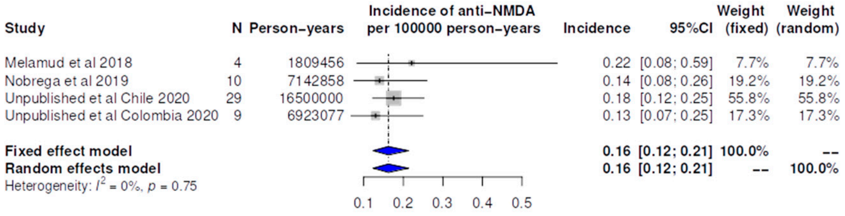

Figure S5. Forest plot summarizing the estimates for the population-based incidence of anti-NMDAR encephalitis in South American countries included in the meta-analysis

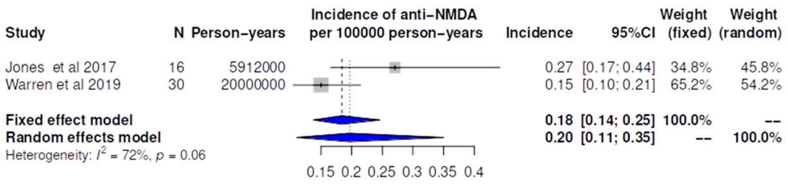

Figure S6. Forest plot summarizing the estimates for the population-based incidence of anti-NMDAR encephalitis in Oceania countries included in the meta-analysis

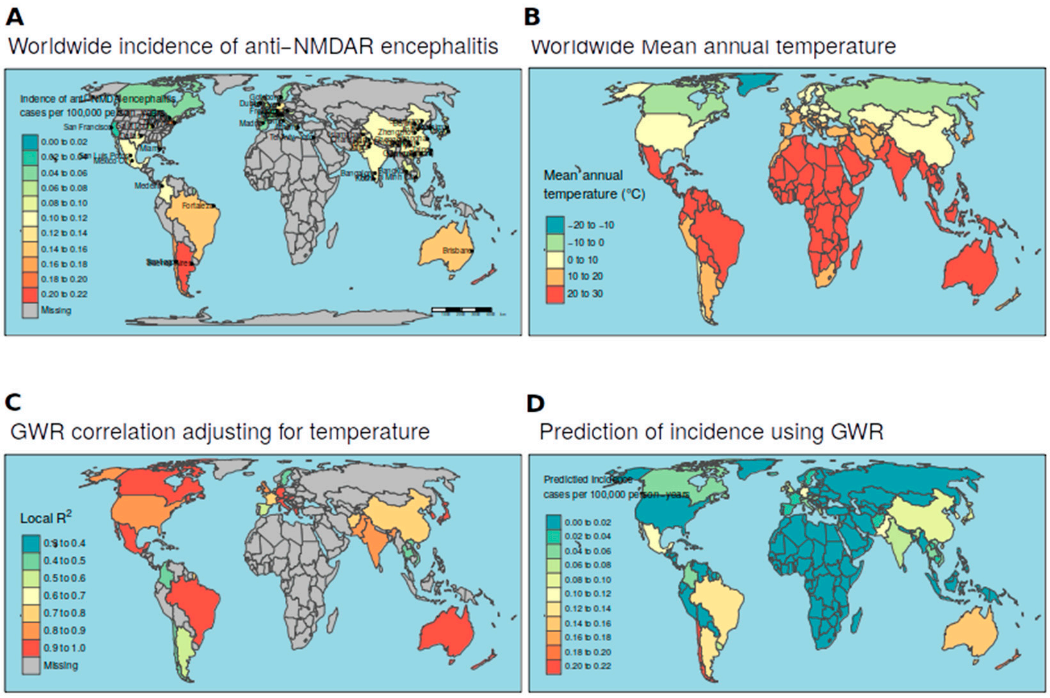

Figure S7. Panel A showing the spatial distribution of anti-NMDAR encephalitis worldwide, using data from countries included in the meta-analysis. Panel B Distribution of the annual mean temperature worldwide. Panel C. Local R² spatial distribution of the incidence of anti-NMDAR encephalitis using GWR model adjusted by the mean annual temperature. Panel D. Prediction of the incidence of anti-NMDAR encephalitis using the GWR model

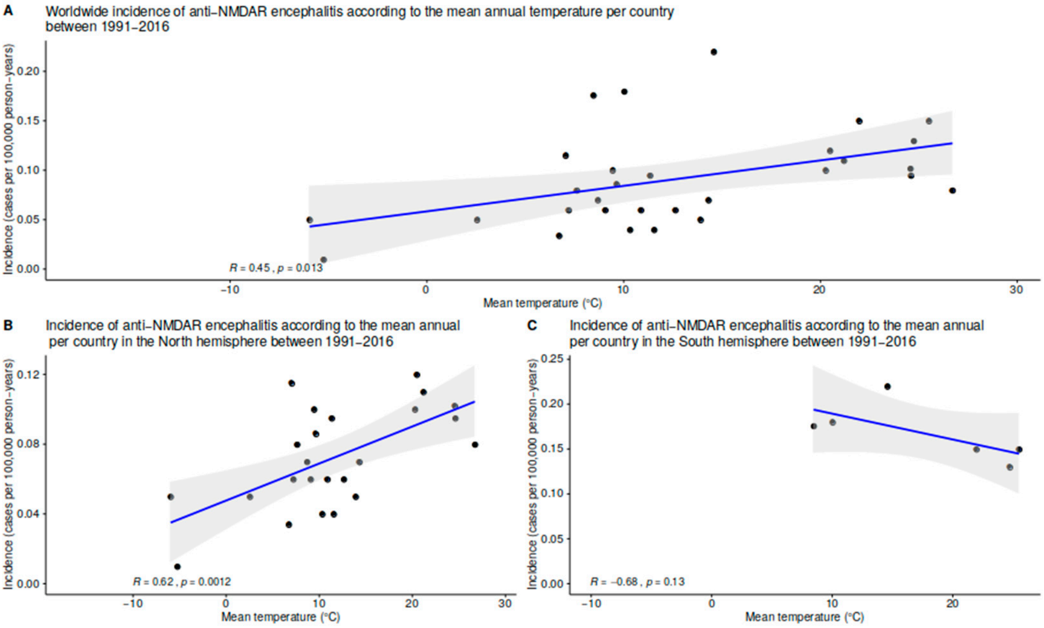

Figure S8. Linear regressions assessing the association between the mean annual temperature at each country and the incidence of anti-NMDAR encephalitis from the meta-analysis, worldwide (A); in the northern hemisphere (B) and the southern hemisphere (C). The grey band around the regression line represents the 95% CI. The R is estimated using the Pearson correlation

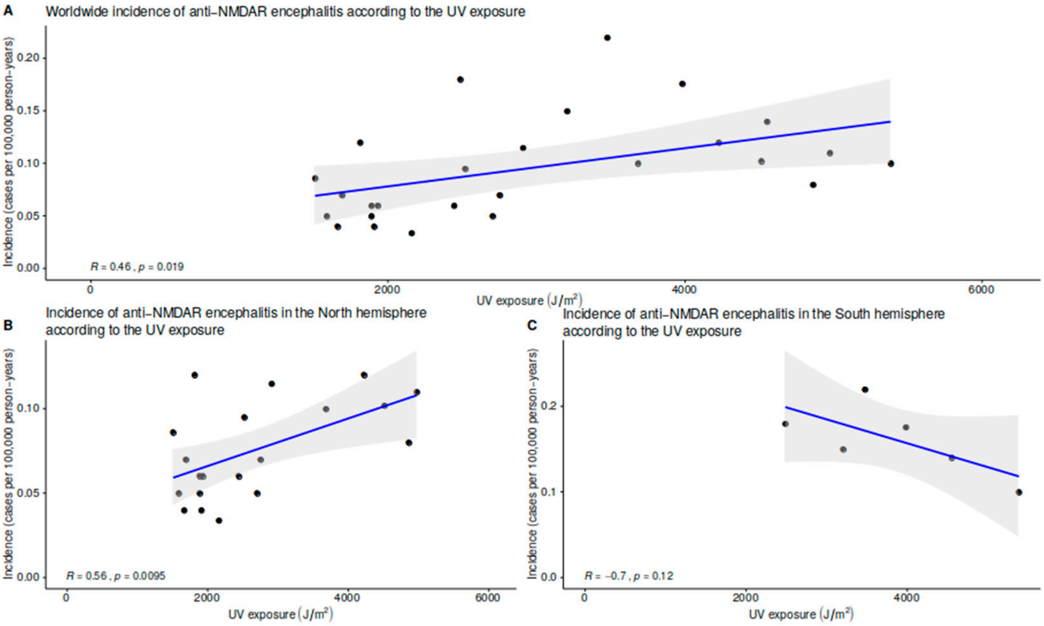

Figure S9. Linear regressions assessing the association between the exposure to ultraviolet radiation exposure of each country and the incidence of anti-NMDAR encephalitis from the meta-analysis, worldwide (A); in the northern hemisphere (B) and the southern hemisphere (C). The grey band around the regression line represents the 95% CI. The R is estimated using the Pearson correlation

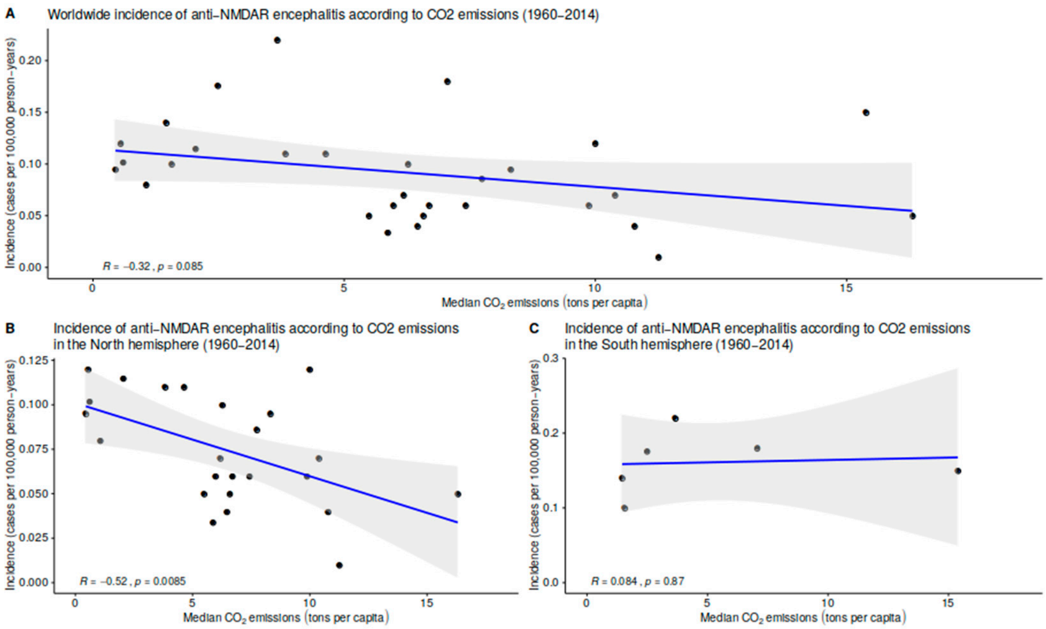

Figure S10. Linear regressions assessing the association between the exposure to CO_2 emissions of every country and the incidence of anti-NMDAR encephalitis from the meta-analysis, worldwide (A); in the northern hemisphere (B) and the southern hemisphere (C). The grey band around the regression line represents the 95% CI. The R is estimated using the Pearson correlation

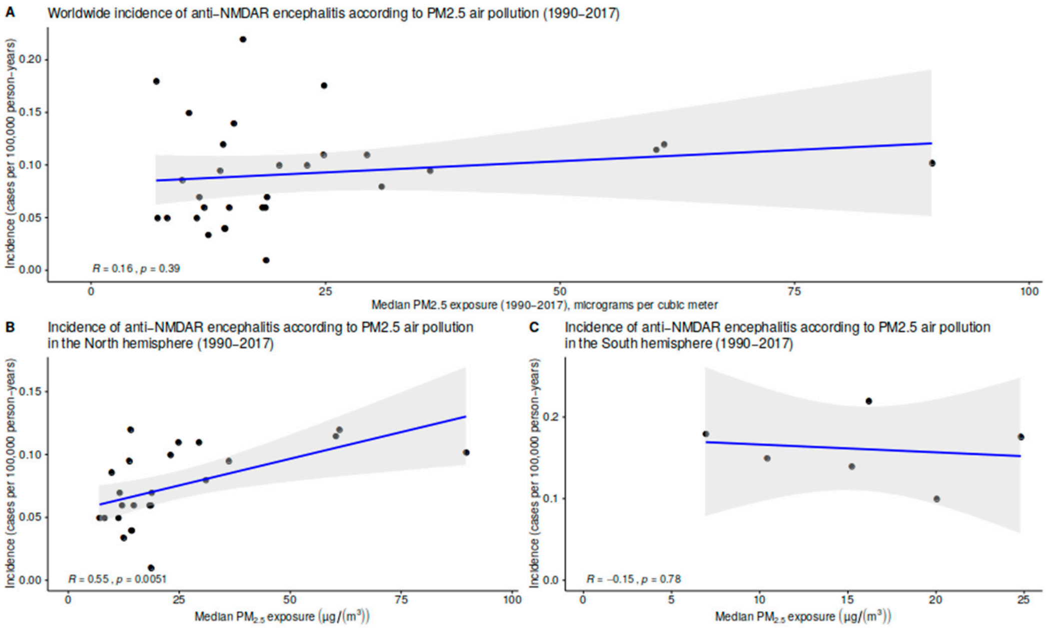

Figure S11. Linear regressions assessing the association between the exposure to PM2.5 air pollution at each country and the incidence of anti-NMDAR encephalitis from the meta-analysis, worldwide (A); in the northern hemisphere (B) and the southern hemisphere (C). The grey band around the regression line represents the 95% CI. The R is estimated using the Pearson correlation

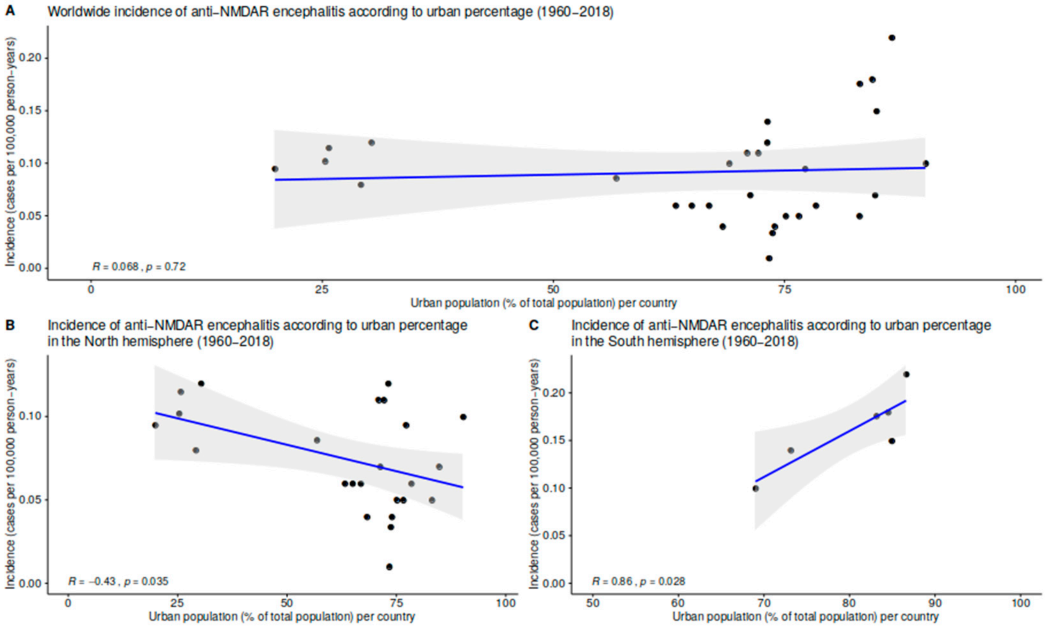

Figure S12. Linear regressions assessing the association between the urban percentage of every country and the incidence of anti-NMDAR encephalitis from the meta-analysis, worldwide (A); in the northern hemisphere (B) and the southern hemisphere (C). The grey band around the regression line represents the 95% CI. The R is estimated using the Pearson correlation

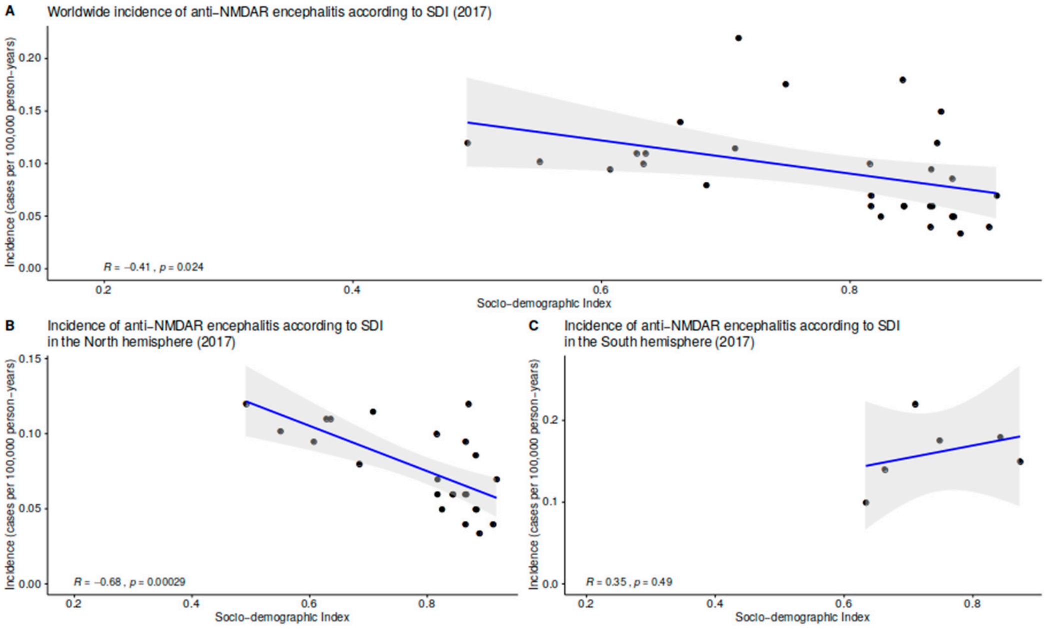

Figure S13. Linear regressions assessing the association between the socio-demographic index (SDI) of every country and the incidence of anti-NMDAR encephalitis from the meta-analysis, worldwide (A); in the northern hemisphere (B) and the southern hemisphere (C). The grey band around the regression line represents the 95% CI. The R is estimated using the Pearson correlation

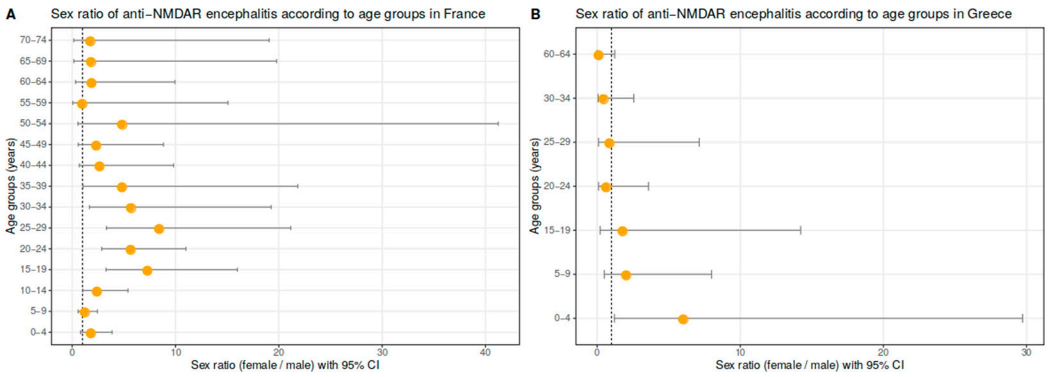

Figure S14. Female/male ratios according to 5-year interval in the French dataset (A) and the Greek dataset (B). The dot represent the rate and the bars are the 95% confidence interval

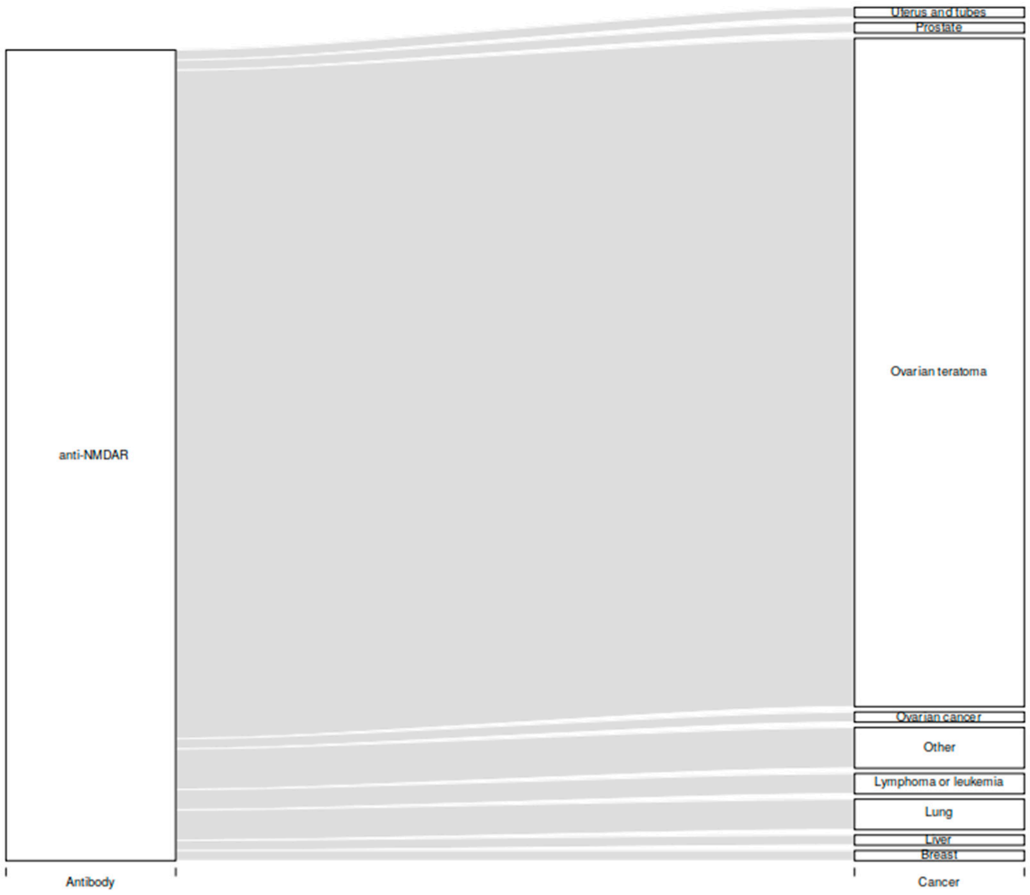

Figure S15. Sankey plot with the anti-NMDAR encephalitis associated with a tumor. The different types of tumors are indicated in the column at the left side and the widths of the bands are linearly proportional to the number of cases.
